# Prognostic value of blood gas parameters and end-tidal carbon dioxide values in out-of-hospital cardiopulmonary arrest patients

**DOI:** 10.3906/sag-1812-156

**Published:** 2019-10-24

**Authors:** Ayça ÇALBAY, Zeynep ÇAKIR, Atıf BAYRAMOĞLU

**Affiliations:** 1 Emergency Department, Erzurum Regional Training and Research Hospital, University of Health Sciences, Erzurum Turkey; 2 Emergency Department, Faculty of Medicine, Atatürk University, Erzurum Turkey

**Keywords:** Arteriol/alveolar carbon dioxide difference, blood gas, end-tidal carbon dioxide, out of hospital cardiopulmonary arrest, resuscitation

## Abstract

**Background/aim:**

This study aimed to evaluate the usefulness of blood gas and end-tidal carbon dioxide (EtCO2) measurements for predicting return of spontaneous circulation (ROSC) and for evaluating post-ROSC neurological survival.

**Materials and methods:**

This was a prospective case control study utilizing Atatürk University’s database of adult nontraumatic patients (over 18 years old) with out-of-hospital cardiac arrest (OHCA) over the course of a year. The neurological status of the patients was evaluated after 1 h at ROSC and at hospital discharge, as defined by the cerebral performance category score. The blood gas parameters pH, PO2, PCO2, lactate, and BE were compared with EtCO2 from capnography and arteriol/alveolar carbon dioxide difference (AaDCO2) by using both blood gas and capnography upon admission to the emergency department and at ROSC.

**Results:**

A total of 155 patients were included in the study to form the control group with ROSC. The PO2, PCO2, and AaDCO2 values showed a prognostic marker for the supply of ROSC (P < 0.05). The EtCO2, lactate, and BE values measured by the blood gas were found to be insignificant in the prediction of ROSC (P > 0.05). Conversely, AaDCO2 was found to be significant in ROSC estimation (P < 0.05), but not in neurological evaluation (P > 0.05).

**Conclusion:**

Blood gas parameters and EtCO2 are sufficient in predicting ROSC. The value of AaDCO2 calculated using EtCO2 and PO2 may be used in predicting the prognosis of OHCA patients, but this value does not provide any conclusions concerning neurological survival.

## 1. Introduction 

### 1.1. Background

Mortality rates after sudden cardiac arrest are high, even when return of spontaneous circulation (ROSC) is provided [1,2]. Arrest interventions aim at ensuring ROSC and minimizing neurological damage after ROSC. Many prognostic factors exist, such as cardiac output, ventilation status, and tissue perfusion; these are closely related to the discharge of cardiac arrest patients. Elevated mortality after ROSC may be associated with postcardiac arrest syndrome, including anoxic neurological damage, myocardial dysfunction, and systemic ischemic/reperfusion response [3]. 

### 1.2. Goals of this investigation

The first aim of the study was to investigate whether arteriole/alveolar carbon dioxide difference (AaDCO2), which is calculated using blood gas parameters, and end-tidal carbon dioxide (EtCO2), which is measured by capnography, could be used as prognostic markers for patients with cardiac arrest in which ROSC is provided. The second aim was to examine whether these markers played a role in short-term neurological evaluation.

## 2. Methods

### 2.1. Study design and setting

This was a prospective case control clinical study of out-of-hospital cardiac arrest (OHCA) patients’ prognostic value on ROSC and neurological recovery by blood gas analyses and EtCO2 values. Patients who were admitted to the clinic’s emergency department with cardiopulmonary arrest from February 2016 to February 2017 were enrolled in the study. Study approval was obtained from the coordinating center’s Institutional Editorial Board with a waiver of informed consent.

### 2.2. Patient selection

Patients were included in the study who were 18 years of age or older and were nontraumatic patients admitted to the emergency department with OHCA. Despite the planned inclusion of pregnant arrest patients, pregnant cardiopulmonary arrest patient entry into the clinic did not occur during the study’s time interval. 

### 2.3. Measurements and variables

Patients were taken to the resuscitation room after emergency department entry. Monitorization and heart rate monitoring were initiated with supraglottic airway supplies, such as balloon-masked ambu, until oxygen was provided with an endotracheal tube (ETT). Simultaneous chest compressions were started. The blood gas sample was then taken with a suitable heparinized syringe at the same time as EtCO2 measurement. The samples were sent to the laboratory in 2 min to prevent the blood from clotting. In the blood gas analysis, pH, PO2, PCO2, lactate, and BE values were measured. The same procedure was repeated again after the patient’s ROSC was obtained. Resuscitation was applied according to the 2015 AHA guidelines until ROSC was provided. After ROSC was provided, the cerebral performance category (CPC) score was noted, during the first hour and at discharge, for neurological evaluation. 

### 2.4. Biochemical analysis

EtCO2 values from the patient and control groups were measured after admission to the hospital by capnography without stopping resuscitation. Simultaneously, blood was drawn to analyze the femoral artery with the help of a heparinized blood gas injector. No medication was given that could affect blood gas parameters of the patients, such as NaHCO3. After the air inside the injector was thoroughly evacuated, the sample was delivered to University Research Hospital Central Laboratories at room temperature with the help of the hospital pneumatic system. If the system was not available, the sample was delivered by the emergency staff. After the blood reached the laboratory, it was taken to the device to be analyzed quickly. Within 3 to 5 min, the analysis results were available. 

PCO2, PO2, pH, lactate, and BE levels from the blood samples were measured using the ABL 800 Basic Auto check radiometer, according to the directions provided by the manufacturer. For patients with ROSC, the same procedure was repeated again within 5 min of the patient’s pulse and rhythm control. 

### 2.5. Statistical analysis 

Statistical analysis of the study was performed with SPSS 20.0 (SPSS Inc, Chicago, IL, USA) and Med Calc for sensitivity, specificity, and positive and negative likelihood ratios. The results were expressed as percentages for categorical variables and as medians (interquartile ranges), mean ± standard deviation, minimum, and maximum for the continuous variables. Categorical data were compared using the chi-square test. The Saphiro–Wilk test was used to evaluate normality with a significance level of 0.05. Continuous variables were compared using the independent sample t-test or the Mann–Whitney U test, as appropriate. Univariate and multivariate regression analyses were performed to investigate the relationship between significant parameters and ROSC. The predictive significance of variables were measured with receiver operating characteristic (ROC) curves, namely PO2, PCO2, and AaDCO2 with cardiopulmonary resuscitation (CPR) before the EMS team reached the hospital, by emergency physicians in the hospital, and by adrenaline doses. The sensitivity, specificity, and positive and negative likelihood ratios (+LR and –LR) were calculated for cutoff values. A value of P < 0.05 was considered statistically significant

## 3. Results

During the course of the study, 249 cardiopulmonary arrest patients came to the emergency department. Of these patients, 94 were traumatic arrest patients and were excluded from the study. A total of 155 outpatients, who were nontraumatic and over 18 years old, were included in the study. 

The blood gases, EtCO2, and AaDCO2 values before and after the resuscitation of the patients are shown in Table 1. When the relationship between blood gas parameters and the values of EtCO2 and AaDCO2 were investigated, in terms of the availability of ROSC and short-term neurological evaluation, PO2 (P = 0.0006), PCO2 (P = 0.0012), and AaDCO2 (P = 0.0003) were found to be statistically significant. 

**Table 1:** Values of blood gases, EtCO2, and AaDCO2 before and after the resuscitation of the patients.

	ROSC	EXİTUS	P-value
Age (year) Median Percentiles 25 Percentiles 75	655578	67.5058.2576.75	0.312
A-etCO2 (mmHg) Median Percentiles 25 Percentiles 75	14.51026.5	127.2517	0.005
A-ph Median Percentiles 25 Percentiles 75	6.976.877.11	6.996.877.12	0.721
A-pCO2 (mmHg) Median Percentiles 25 Percentiles 75	54.5541.373.27	68.555.887.72	0.0012
A-pO2 (mmHg) Median Percentiles 25 Percentiles 75	51.528.273.45	26.8518.6553.25	0.0006
A-BE Median Percentiles 25 Percentiles 75	–15.95–21.75–11.25	–12.8–18.75–8.7	0.155
A-Lactat (mmol/L) Median Percentiles 25 Percentiles 75	11.69.9514.7	12.48.816	0.484
A-AapCO2 (mmHg) Median Percentiles 25 Percentiles 75	38.522.3556.75	54.441.672.2	0.0003
B-ECPR (minute) Median Percentiles 25 Percentiles 75	5310	10520	0.0006
B-HCPR (minute) Median Percentiles 25 Percentiles 75	3010	10520	<0.0001
HCPR (minute) Median Percentiles 25 Percentiles 75	201035	453550	<0.0001
Adrenalin Time (x1mg) Median Percentiles 25 Percentiles 75	539	10915	<0.0001
Defibrilation time Median Percentiles 25 Percentiles 75	003	003	0.773
Amiodaron Doze (mg) Median Percentiles 25 Percentiles 75	00450	00450	0.902
NaHCO3 Doze (x8,4%NaHCO3=1ml/kg) Median Percentiles 25 Percentiles 75	000	000	0.225
O2 (lt/dk) Median Percentiles 25 Percentiles 75	151015	131015	0.394
R-etCO2 (mmHg) Median Percentiles 25 Percentiles 75	2620.7537.75	-	-
R-ph Median Percentiles 25 Percentiles 75	6.986.857.06	-	-
R-pCO2 (mmHg) Median Percentiles 25 Percentiles 75	61.141.975.55	-	-
R-PO2 (mmHg) Median Percentiles 25 Percentiles 75	79.853.75129	-	-
R-BE Median Percentiles 25 Percentiles 75	–15.1–19.2–9.95	-	-
R-Lactat (mmol/L) Median Percentiles 25 Percentiles 75	11.5914.15	-	-
R-AapCO2 (mmHg) Median Percentiles 25 Percentiles 75	32.613.546.8	-	-

A-EtCO2: admission endtidal carbon dioxide, A-ph: admission ph, A-PCO2: admission PCO2, A-PO2: admission PO2, A-BE: admission BE, A- lactat: admission lactat, A-AaDCO2: admission AaDCO2, R-EtCO2: ROSC EtCO2, R-ph: ROSC ph, R-PCO2: ROSC PCO2, R-O2: ROSC O2, R-BE: ROSC BE, R-lactat: ROSC lactat, R-AaDCO2: ROSC AaDCO2, ROSC: return of spontaneous circulation

The sensitivity, specificity, positive, and negative predictive values, and positive and negative likelihood ratios of all useful values are shown in Table 2. The same table shows the importance of CPR before EMS providers arrived (P = 0.0006), in the ambulance (P < 0.0001), in the hospital (P <0.0001), and upon administration of adrenaline (P < 0.0001).

**Table 2:** The sensitivity, specificity, positive and negative predictive values, and positive and negative likely ratios of significant values.

Parameter	A-PCO2	A-PO2	A-AaDCO2	B-ECPR	B-HCPR	HCPR	Adrenalin Doze
P-value	0.0012	0.0006	0.0003	0.0006	<0.0001	<0.0001	<0.0001
Cutoff	≤53.6	>27.9	≤45.2	≤10	≤2	≤25	≤7
Sensitivity	50	76.92	66.04	83.05	49.15	66.1	69.49
95%CI	36.1–63.9	63.2–87.5	51.7–78.5	71.0–91.6	35.9–62.5	52.6–77.9	56.1–80.8
Specificity	77.78	54.4	68.97	39.58	83.33	91.67	86.46
95%CI	67.8–85.9	43.6–65	58.1–78.5	29.7–50.1	74.4–90.2	84.2–96.3	78–92.6
+LR	2.25	1.69	2.13	1.37	2.95	7.93	5.13
95%CI	1.4–3.6	1.3–2.2	1.5–3.1	1.1–1.7	1.8–4.9	4–15.8	3–8.7
–LR	0.64	0.42	0.49	0.43	0.61	0.37	0.35
95%CI	0.5–0.9	0.2–0.7	0.3–0.7	0.2–0.8	0.5–0.8	0.3–0.5	0.2–0.5
+PV	57.4	49.4	56.5	45.8	64.4	83	75.9
95%CI	45.8–68.3	42.7–56.1	47.3–65.2	40.9–50.8	51.9–75.2	71–90.7	64.9–84.3
–PV	72.2	80.3	76.9	79.2	72.7	81.5	82.2
95%CI	66–77.6	70.6–87.4	69.1–83.3	67.2–80.6	67.2–80.6	75.4–86.3	75.7–87.2

A-PCO2: admission PCO2, A-PO2: admission PO2, A-AaDCO2: admission AaDCO2, B-ECPR: CPR before EMS providers, B-HCPR: CPR in ambulance, HCPR: CPR in hospital

## 4. Discussion

The prognostic value of blood gas analyses, EtCO2 values, and AaDCO2 values for the ROSC and short-term neurological recovery were prospectively analyzed.

Acidosis, peripheral vasodilation, reduction of the preload of the heart, and inability to effectively perform CPR increased PCO2. Reasons for this value can be both patient-originated and nonpatient-originated. Therefore, the use of PCO2 with a value such as EtCO2 might strengthen the ROSC estimate [4]. In this study, the value of PCO2 measured from the bloodstream was in agreement with previous studies and could be used in predicting the ROSC of the patient (P = 0.0012).

PO2 was not a reliable indicator of tissue hypoxia in blood gases from cardiac arrest patients. This effect was due to arterial, venous, or mix formation and was affected by the intake site of the bloodstream, as well as whether CPR was effective and complete perfusion was not provided. For example, values measured from the fingertip were lower than values measured in the blood gas due to low cardiac output and poor circulation [5]. A statistically significant relationship was established between PO2 values measured from blood gases and ROSC at the time of admission to the emergency clinic. This evaluation showed that PO2 values could be used in ROSC estimation, in agreement with previous studies (P = 0.0006).

Many respiratory, metabolic, and circulatory factors cause the measured lactate value to be high in blood gas analysis. The basis of these values are the onset of anaerobic metabolic activities in tissues experiencing decreased tissue perfusion, increased tissue oxygen demand, and insufficient oxygen during resuscitation of cardiac arrest patients. According to previous studies, a lactate value higher than 2, more pronounced tissue hypoxia, and the presence of 4 or more are indications of higher risk of patient mortality [6]. In this study, the mean lactate levels in arrest patients were measured as 12.7 ± 4.49 (1.6–29), and lactate levels after ROSC administration were measured as 11.78 ± 4.84. The maximum lactate level was 29, and the maximum lactate level of ROSC patients was 24. No significant correlation was found between the elevated prognostic value of the lactate levels and ROSC (P = 0.484), and no statistically significant effect of lactate as a prognostic marker was found.

BE is the amount of acid or base added to bring the pH to 7.40 at 37 °C and 40 mm Hg partial CO2 pressure. The normal value is between –2.5 and +2.5 mmol/L. In this study, the lowest and highest BE values measured were –28 and 17.5, respectively, and the average was –14.37 ± 6.96. Some studies mentioned BE as a predictor of ROSC, but in this study, statistical data to support this was not reached (P = 0.155).

EtCO2 followed by capnography is a useful monitoring option for cardiac arrest patients in which follow-up of patients’ resuscitation can be done. This process has the important advantage of being easy to implement; it does not require experience or special skills for application. It is low-cost and noninvasive. During resuscitation, effective ventilation and effective chest pressure increase the measured EtCO2 value. A sudden increase in the EtCO2 value during resuscitation can be interpreted as providing ROSC [7–9]. In this study, the arrival of the emergency clinic arrest patients with an average of 15.66 ± 10.15 mm Hg EtCO2 average value for patients provided ROSC was measured as the average of 29.48 ± 14.98 mm Hg. The minimum EtCO2 values were 2 mm Hg for the acceptance of arrest patients and 6 mm Hg after ROSC was given. In this study, a statistically significant correlation between EtCO2 and ROSC as shown by the literature was not found (P = 0.005). 

Because resuscitation is a dynamic process, and hardness of arterial blood gas is present in cardiac arrest patients, resuscitation needs more prognostic and neurological indicators independent from patient and devices which cannot be affected PCO2 measurements. The most beneficial methods for the follow-up and prognosis of patients undergoing resuscitation are EtCO2 measured by capnography, and PCO2 and lactate levels measured by blood gas. There has not yet been much research to back the notion that AaDCO2, the difference between PCO2 measured from arterial blood gas and EtCO2 measured by capnography, could be used as a follow-up and prognostic indicator of resuscitated cardiac arrest patients. Some studies have mentioned that the value of AaDCO2 might have an effect on mortality and ROSC and, in some cases, may be indicative of CPR activity and organ perfusion [10, 11]. In this study, the mean AaDCO2 value was calculated as 52.77 ± 30.49 mm Hg (–4.9 to 153 mm Hg) on admission to the emergency clinic, and 6.02 ± 39.73 mm Hg after ROSC admission, parallel to the study by Spindelboeck et al. [12]. The maximum value at admission to the emergency room was 153 mm Hg, and the maximum value after ROSC was given was 148 mm Hg. According to the statistical analysis performed, a significant relationship was found between ROSC and AaDCO2 values, which were calculated when the patients came to the emergency clinic (P = 0.0003). The AaDCO2 values calculated before and after ROSC delivery were found to be useful in predicting the likelihood of ROSC formation.

In this study, CPC was used as the measurement of neurological survival and follow-up of the groups. The CPC value measured during the first hour of patients’ ROSC was defined as the short-term symptomatology, and the CPC value at the patients’ discharge was recorded. The mean CPC of ROSC patients was 4.54 ± 0.7. According to the results of statistical analysis, no significant relationship was found between AaDCO2 values and CPC (P = 0.19).

In conclusion, PCO2, PO2, and AaDCO2 can be used as prognostic markers of OHCA patients returning to spontaneous circulation when they are admitted to emergency services. PCO2 values were calculated from arterial blood gas, and AaDCO2 values were calculated by EtCO2 measured by capnography; these were shown to have a more significant statistical value in the prediction of ROSC. Blood gas parameters and EtCO2 values did not show any activity as neurological improvement markers in the short term, opposite to Hope Kilgannon J et al.’s study [13], and the AaDCO2 value calculated using these 2 assessments showed that neurological improvement could not be used as a prognostic marker.

## References

[ref1] (2010). Predictors of survival from out-of-hospital cardiac arrest: a systematic review and meta-analysis. Circulation: Cardiovascular Quality and Outcomes.

[ref3] (2014). Hospital variation in survival trends for in-hospital cardiac arrest. Journal of the American Heart Association.

[ref5] (2008). Post-cardiac arrest syndrome: epidemiology, pathophysiology, treatment, and prognostication. Circulation.

[ref7] (2016). Role of blood gas analysis during cardiopulmonary resuscitation in out-of-hospital cardiac arrest patients. Medicine (Baltimore).

[ref9] (2014). The use of transcutaneous CO2 monitoring in cardiac arrest patients: a feasibility study. Scandinavian journal of trauma resuscitation and emergency medicine.

[ref11] (2016). Hemodynamic monitoring. Tintinalli’s Emergency Medicine: A Comprehensive Study Guide 8th ed. United States.

[ref13] The determination of end-expiratory CO2 during resuscitation. Experience and results with the Normocap 200 (Fa.

[ref16] (1987). End-tidal carbon dioxide monitoring during cardiopulmonary resuscitation. JAMA.

[ref18] (1996). End-tidal carbon dioxide changes during cardiopulmonary resuscitation after experimental asphyxial cardiac arrest. American journal of emergency medicine.

[ref20] (1997). A comparison of the end-tidal-CO2 documented by capnometry and the arterial pCO2 in emergency patients. Resuscitation.

[ref22] (2005). Correlation of arterial PCO2 and PETCO2 in prehospital controlled ventilation. American journal of emergency medicine.

[ref24] (2016). Arterial blood gases during and their dynamic changes after cardiopulmonary resuscitation: a prospective clinical study. Resuscitation.

[ref26] (2019). Partial pressure of arterial carbon dioxide after resuscitation from cardiac arrest and neurological outcome: A prospective multi-center protocol-directed cohort study. Resuscitation.

